# Computational prediction and molecular confirmation of *Helitron *transposons in the maize genome

**DOI:** 10.1186/1471-2164-9-51

**Published:** 2008-01-28

**Authors:** Chunguang Du, Jason Caronna, Limei He, Hugo K Dooner

**Affiliations:** 1 Dept. of Biology & Molecular Biology, Montclair State University, Montclair, NJ 07043, USA; 2 Waksman Institute, Rutgers University, Piscataway, NJ 08854, USA; 3 Dept. of Plant Biology, Rutgers University, New Brunswick, NJ 08901, USA

## Abstract

**Background:**

*Helitrons *represent a new class of transposable elements recently uncovered in plants and animals. One remarkable feature of *Helitrons *is their ability to capture gene sequences, which makes them of considerable potential evolutionary importance. However, because *Helitrons *lack the typical structural features of other DNA transposable elements, identifying them is a challenge. Currently, most researchers identify *Helitrons *manually by comparing sequences. With the maize whole genome sequencing project underway, an automated computational *Helitron *searching tool is needed. The characterization of *Helitron *activities in maize needs to be addressed in order to better understand the impact of *Helitrons *on the organization of the genome.

**Results:**

We developed and implemented a heuristic searching algorithm in PERL for identifying *Helitrons*. Our HelitronFinder program will (i) take FASTA-formatted DNA sequences as input and identify the hairpin looping patterns, and (ii) exploit the consensus 5' and 3' end sequences of known *Helitrons *to identify putative ends. We randomly selected five predicted *Helitrons *from the program's high quality output for molecular verification. Four out of the five predicted *Helitrons *were confirmed by PCR assays and DNA sequencing in different maize inbred lines. The HelitronFinder program identified two head-to-head dissimilar *Helitrons *in a maize BAC sequence.

**Conclusion:**

We have identified 140 new *Helitron *candidates in maize with our computational tool HelitronFinder by searching maize DNA sequences currently available in GenBank. Four out of five candidates were confirmed to be real by empirical methods, thus validating the predictions of HelitronFinder. Additional points to emerge from our study are that *Helitrons *do not always insert at an AT dinucleotide in the host sequences, that they can insert immediately adjacent to an existing *Helitron*, and that their movement may cause changes in the flanking region, such as deletions.

## Background

*Helitrons *represent a new class of transposable elements recently uncovered in animals and plants [1], including maize [2-4]. The first two *Helitrons *described in maize were the causative agents of stable mutations: one in the *shrunken2 *mutant *sh2-7527 *[2] and another one in the *barren stalk1 *reference mutant *ba1-Ref *[3]. The termini of a 6525-bp *Helitron *in the *ba1-Ref *mutant share striking similarity with those of the *Helitron *insertion in the *sh2-7527 *mutant, indicating that they belong to the same family. Lai et al. [4] reported that two *Helitrons*, *HelA *and *HelB*, accounted for all of the genic differences distinguishing two previously described *bz *locus haplotypes [5]. *HelA *is 5.9-kb long and contains sequences for three of the four genes found only in the McC *bz-locus *haplotype. A nearly identical copy of *HelA *was isolated from a different chromosomal site in the B73 inbred. Both sites appear to be polymorphic in maize, suggesting that these *Helitrons *have been active recently.

Basic *Helitron *features include:

• Conserved TC and CTAG sequences at the 5' and 3' termini, respectively

• Palindromes (16- to 20-bp 'hairpin loops') 10–15 bp upstream of the 3' terminus

• Flanking A and T host nucleotides at the 5' and 3' termini, respectively

**Figure 1:**
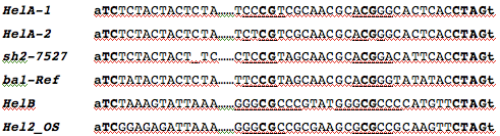
*Helitron *end sequence alignment by Lai et al. [4]. It contains the 5' and 3' termini of the maize *Helitrons HelA-1 *and *HelB *from line McC, *HelA-2 *from B73, the *Helitron *insertions in mutants *sh2-7523 *and *ba1-Ref*, and the rice *Helitron2_OS*. *Helitron *sequences are in uppercase letters and the invariant host nucleotides where the *Helitrons *insert are in lowercase letters. Conserved nucleotides at the 5' and 3' termini are in bold uppercase letters and the inverted repeats at the 3' termini are underlined. The nonconserved body of the *Helitrons *is represented by dots.

The Figure 1 of a recent paper [4] comparing *Helitron *end sequences contains the 5' and 3' termini of the maize *Helitrons HelA-1 *and *HelB *from line McC, *HelA-2 *from B73, the *Helitron *insertions in mutants *sh2-7523 *and *ba1-Ref*, and the rice *Helitron2_OS*. *Helitron *sequences are in uppercase letters and the invariant host nucleotides where the *Helitrons *insert are in lowercase letters. Conserved nucleotides at the 5' and 3' termini are in bold uppercase letters and the inverted repeats at the 3' termini are underlined. The nonconserved body of the *Helitrons *is represented by dots.

Besides the typical *Helitron *features they all share, there are two invariant CGs located 10 bp apart in each member of the palindromic repeat, the second one occurring just 9 bp from the 3' end. In the *HelA *subgroup, there is an invariant AA dinucleotide between the palindromic repeats. The 3' terminal 30 bp of *HelA *are very conserved with other *Helitrons*. In fact, of those 30 bp, *HelA *shares 26 and 24 bp, respectively, with the *Helitrons *previously identified as the causative agents of mutations at *sh2 *and *ba1*.

One remarkable feature of *Helitrons *is their ability to capture gene sequences, a feature that makes them of considerable potential evolutionary importance. However, because *Helitrons *lack the typical structural features of other DNA transposable elements, identifying them is a challenge. Currently, most researchers identify *Helitrons *manually by comparing sequences. For example, Wang and Dooner [6] identified *Helitrons *by vertical comparisons of the *bz *regions from 8 different maize inbred lines. Although very precise, this approach is time consuming. Just lately, one model-based identification of *Helitrons *was introduced for *Arabidopsis thaliana *[7]. With the maize whole genome sequencing project underway, an automated computational *Helitron *searching tool is needed. The characterization of *Helitron *activities in the maize genome needs to be addressed in order to better understand the impact of *Helitrons *on the organization of the maize genome.

## Results

### Identification of *Helitrons *by *in silico *Analysis

There are basically two main non-autonomous categories of *Helitrons *in maize, *Hel1 *or *HelA*, and *Hel2 *or *HelB*. The majority of identified *Helitrons *in maize are of the *HelA *type (listed in Table 1, which was kindly provided by Dr. S. Lal), so our HelitronFinder program is focussed exclusively on the prediction of maize *HelA *type *Helitrons*.

**Table 1:** Known *HelA *Type *Helitrons *in Maize

**Helitron**	**Maize line**	**Accession**	**Start**	**End**	**Size**	**Source**
*HelA-1b*	W22	DQ186636	1	5189	5189	He & Dooner, 2005 [10]
*HelA-1c*	W22	DQ186637	1	5189	5189	Li & Dooner, 2005 [11]
*Hel1-1*		AF293457	1	~	17700	Lal et al., 2003 [2]
*Hel1-2*		AY645947	1	6525	6525	Gupta et al., 2005 [3]
*Hel1-3a*	B73	AF46693	48370	82950	34581	Gupta et al., 2005 [3]
*Hel1-3b*	B73	AF466932	38471	73780	35310	Gupta et al., 2005 [3]
*Hel1-4*	BSS53	AF090447	4408	22158	17751	Gupta et al., 2005 [3]
*Hel1-5a*	McC	DQ186635	1	5858	5858	Lai et al., 2005 [4]
*Hel1-7a*	B73	AY664413	210885	205938	4946	Morgante et al., 2005 [5]
*Hel1-7c*	Mo17	DQ002408	47752	56904	9153	Brunner et al, 2005 [12]
*Hel1-7d*	Mo17	DQ002406	61262	66313	5052	Brunner et al, 2005 [12]
*Hel1-8*	B73	AY664413	240549	259755	19207	Morgante et al., 2005 [5]
*Hel1-9*	B73	AY664413	7748	5070	2677	Morgante et al., 2005 [5]
*Hel1-10*	B73	AY664414	89533	81613	7919	Morgante et al., 2005 [5]
*Hel1-12*	B73	AY371488	96529	89735	6793	Morgante et al., 2005 [5]
*Hel1-13*	B73	AY530951	134622	138054	3433	Morgante et al., 2005 [5]
*Hel1-14*	B73	AY664419	262092	273049	10958	Morgante et al., 2005 [5]
*Hel1-15*	B73	AY664415	266036	267537	1502	Morgante et al., 2005 [5]

All the *Helitrons *in this table, which was kindly provided by Dr. S. Lal, have been published. The pertinent references are listed under the "Source" column. The accession numbers refer to entries in the GenBank sequence database: the *Helitron *coordinates in the sequence are identified under the "Start" and "End" columns.

**Figure 2:**
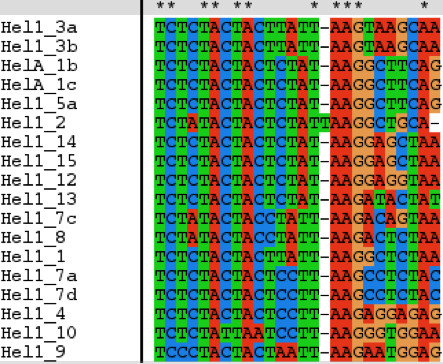
Alignment of the first 25 nucleotides of known maize *Helitron *5' ends. A * means that all the sequences at that particular location are the same. There is a strong similarity in the first 18 nucleotides among the aligned *Helitrons*. The consensus from the alignment is our main criterion to search for the 5' end of new *Helitrons*.

The 'hairpin loop' and the CTAG termini at the 3' end of known *Helitrons *are the key characteristics for the identification of new *Helitrons*. The most challenging part is to identify the 5' end. For this purpose, we selected the first 25 nucleotides from the 5' end of each known *Helitron *of Table 1 and aligned them using Clustal [8]. There is a strong similarity in the first 18 nucleotides among the aligned *Helitrons *(Fig. 2). The consensus from the alignment is our main criterion to search for the 5' end of new *Helitrons*.

**Figure 3:**
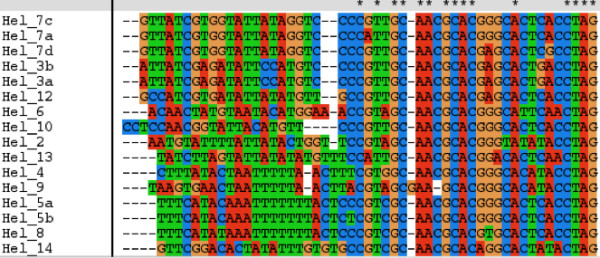
Alignment of the last 50 nucleotides of known maize *Helitrons *3' end. A * means that all the sequences at that particular location are the same. The 3' ends of known *Helitrons *have CTAG termini. For *HelA *type *Helitrons*, the double 'A' is often in the middle of the 'CG' bases in the hairpin loop.

**Figure 4:**
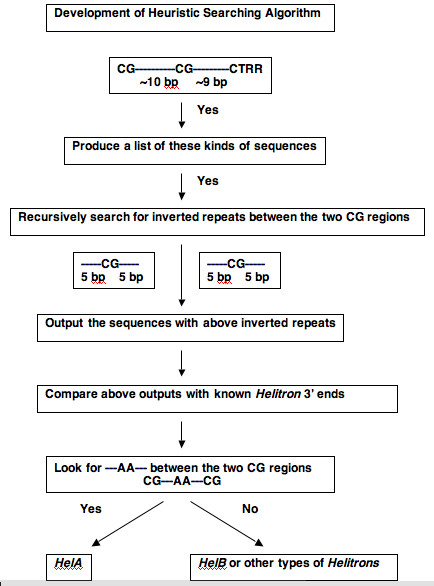
The heuristic algorithm for searching 3' end of *Helitrons*. The 'hairpin loop' and the CTAG termini at the 3' end of known *Helitrons *are the key characteristics for the identification of new *Helitrons*. For *HelA *type *Helitrons*, the double 'A' is often in the middle of the 'CG' bases in the hairpin loop.

We chose the first 18 nucleotides from Figure 1 as our 5' end search criterion:

TC [TC] [CA]TA [CT]TA [CA] [TC] [TCA] [TA] [T or none]AAG. Ambiguous nucleotides at a particular location are included within brackets []. The 3' ends of known *Helitrons *have CTAG termini. For *HelA *type *Helitrons*, the double 'A' is often in the middle of the 'CG' bases in the hairpin loop (Fig. 3). The approaches used for searching 3' ends are detailed in figure 4.

We downloaded maize sequences from the GenBank non-redundant database to our local Sun workstation and used the HelitronFinder program to predict *Helitron *candidates. There are 44 and 102 predicted *Helitrons *in our "high quality" and "medium quality" outputs, respectively. The output files are in text format, with a GenBank accession number for each predicted *Helitron*. Outputs specifically identify *Helitron *sequences as being in a forward or reverse complement orientation. The HelitronFinder program also successfully identified all the known *Helitrons *listed in Table 1.

### Confirmation of *Helitrons *by Molecular Analysis

We randomly selected five predicted *Helitrons *from the program's high quality output for molecular verification. PCR primers were designed based on the flanking sequence of each predicted *Helitron*. We surveyed 11 maize inbred and genetic lines for three of the five *Helitron *candidates and 15 lines for the other two. Four sets of primers successfully amplified either the *Helitron*-occupied or the *Helitron-*vacant site from different lines. The PCR products highlighted in bold in Table 2 were cloned and sequenced for further confirmation.

**Table 2:** Molecular Verification of *Helitrons*

**Germplasm**	**Silico 1**	**Silico 2**	**Silico 3**	**Silico4**
4Co63	**Vacant**	Occupied	x	**Vacant**
A188	x	x	x	**Vacant**
A636	**Vacant**	**Occupied**	**Occupied**	**Vacant**
B73	**Vacant**	**Occupied**	**Occupied**	**Vacant**
BSSS53	**Occupied**	x	x	**Vacant**
McC	**Vacant**	**Occupied**	**Vacant**	Occupied
H99	**Vacant**	**Vacant**	x	**Vacant**
M14	**Vacant**	Occupied	x	**Occupied**
Mo17	**Vacant**	**Vacant**	x	Vacant
W22	**Vacant**	Occupied	**Vacant**	x
W23	**Occupied**	x	**Vacant**	Occupied
CML139			x	**Vacant**
I137 TN			x	**Vacant**
Ki3			x	x
bz-R			Vacant	**Vacant**

Silico 1, Silico 2, Silico 3, and Silico 4 are *Helitron *candidates predicted by the HelitronFinder program. 4Co63, A188, A636, B73, BSSS53, H99, M14, Mo17, W22, W23, CML139, I137 TN, and Ki3 are inbred lines. McC and *bz-R *are genetic lines. Vacant: amplified PCR product lacks a *Helitron*.

Occupied: amplified PCR product contains a *Helitron*, which was confirmed by sequencing.

X: no PCR product detected.

Blank: line not tested for the corresponding *Helitron *candidate.

The PCR products highlighted in yellow have been cloned and sequenced for further confirmation.

Four out of five selected predicted *Helitrons *were confirmed by PCR products from different maize inbred lines (Table 2). They are named Silico 1, 2, 3, and 4, and are predicted from BSSS53, B73, B73, and McC sequences, respectively. The "occupied" and "vacant" entries denote PCR bands corresponding to the presence and absence of *Helitrons*, respectively. The X sign stands for no PCR amplification product. In addition to the inbreds from whose sequences they were predicted, *Helitrons *were detected in other inbreds. Thus, Silico 1 is present in W23, besides BSSS53, but absent in eight other inbred lines; Silico 2 is present in 4Co63, A636, McC, M14, and W22, besides B73, but absent in H99 and Mo17; Silico 3 is present in A636, besides B73, but absent in McC, W22, W23, and a *bz-R *genetic line, and Silico 4 is present in M14 and W23, besides McC, but absent in 4Co63, A188, A636, B73, H99, Mo17, CML139, I137TN, and *bz-R*.

**Figure 5:**
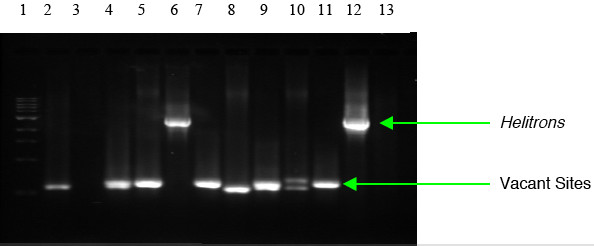
Silico1 PCR products. Lanes: 1, size markers; 2, 4Co63; 3, A188; 4, A636; 5, B73; 6, BSSS53; 7, McC; 8, H99; 9, M14; 10, Mo17; 11, W22; 12, W23; 13, H_2_O. Silico1 is predicted from BSSS53 via our HelitronFinder software and is underlined in order to differentiate it from other lines. A *Helitron*-occupied site was also detected in W23 while *Helitron*-vacant sites were detected in 4Co63, A636, B73, McC, H99, M14, Mo17, and W22.

Silico1 was predicted from the BSSS53 sequence. A *Helitron*-occupied site was also detected in W23 while *Helitron*-vacant sites were detected in 4Co63, A636, B73, McC, H99, M14, Mo17, and W22 (Fig. 5). This result reveals +/- polymorphism among different inbred lines and confirms that the predicted *Helitron*, Silico1, is genuine.

**Figure 6:**
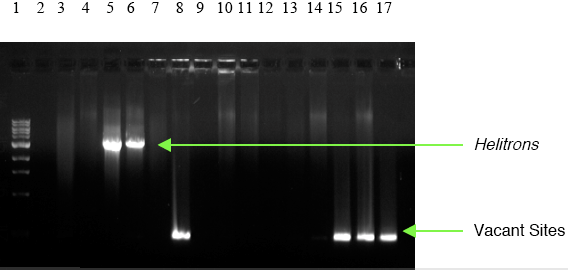
Silico 3 PCR products. Lanes: 1, size markers; 2, blank; 3, 4Co63; 4, A188; 5, A636; 6, B73; 7, BSSS53; 8, McC; 9, CML139; 10, H99; 11, I137TN; 12, Ki3; 13, M14; 14, Mo17; 15, W22; 16, W23; 17, *bz-R*. Silico 3 is predicted from B73 via our HelitronFinder software and is underlined in order to differentiate it from other lines. Both B73 and A636 show *Helitron *occupied sites, whereas lines McC, A188, W22, W23, and *bz-R *show *Helitron *vacant sites. In addition to the *Helitron *band amplified from B73, there was a faint band of the same size as the vacant site. We sequenced this product and confirmed it to be a vacant site.

Silico3 was predicted from the B73 maize sequences. A total of 15 maize lines were used for molecular verification of this HelitronFinder prediction (Fig. 6). Both B73 and A636 show *Helitron *occupied sites, whereas lines McC, A188, W22, W23, and *bz-R *show *Helitron *vacant sites. In addition to the *Helitron *band amplified from B73, there was a faint band of the same size as the vacant site. We cloned and sequenced this product and confirmed it to be a vacant site.

### Characterizations of *Helitrons *in the Maize Genome

#### Discovery of Two Adjacent Helitrons

**Figure 7:**
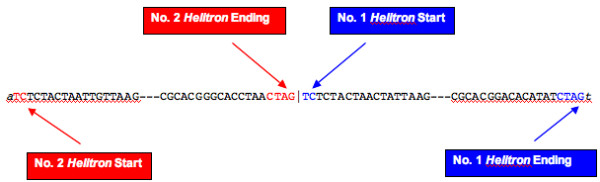
Two adjacent *Helitrons *detected in the *r1 *region of B73 (GenBank accession number AF466202).

The HelitronFinder program identified two adjacent, head-to-tail *Helitrons *in a maize BAC sequence with GenBank accession number AF466202 (Fig. 7). This is the first case of back-to-back *Helitrons *detected in the maize genome. A peculiarity of these head-to-tail *Helitron *configurations is that the TC 5' terminus of the second *Helitron *follows the CTAG 3' terminus of the first, creating a novel G/T junction, rather than the A/T junction normally found at a *Helitron*'s 5' end. Pritham and Feschotte [9] reported several cases of perfect head-to-tail junctions of two *Helitron *elements in the genome of the bat *Myotis lucifugus*. They suggested that these were tandem repeats of *Helitrons *in the *Myotis lucifugus *genome. They also argued that one would expect the A of the host target site to occur between the CTAG end of the first element and the TC start of the second element if the elements had inserted independently. We aligned these two adjacent maize *Helitrons *and found that the sequences differed significantly and contained different genes or gene fragments. This indicates they are not tandem repeats, but arose by consecutive insertions.

**Figure 8:**
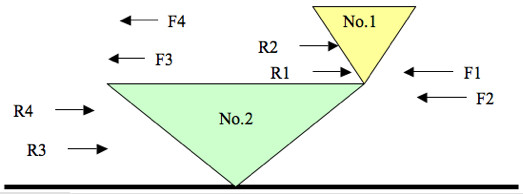
Location of PCR primers flanking and internal to adjacent *Helitrons *identified in sequence AF466202. We designed four pairs of primers for these two *Helitrons*: F1/R1, F3/R3, F2/R4, and F4/R4. F and R represent forward and reverse primers, respectively.

We designed four pairs of primers for these two *Helitrons*, F1/R1, F3/R3, F2/R4, and F4/R4 (Fig. 8). F and R represent forward and reverse primers, respectively. According to the PCR products in Table 3, we detected both *Helitrons *in lines A636 and B73, only *Helitron *No.2 in lines McC, W22, and W23, and neither *Helitron *in lines A188, CML139, H99, Ki3, M14, or Mo17. This result lends itself to two interpretations. One possibility is that *Helitron *No.2 (left) inserted into the maize genome first and that *Helitron *No.1 (right) inserted subsequently, and noncanonically, at the GT dinucleotide found at the 3' end of *Helitron *No.2. An alternative is that *Helitron *No.1 inserted first and *Helitron *No.2 inserted subsequently, and canonically, at the AT dinucleotide created by the host A and the T at the 5' end of *Helitron *No.1. Following the formation of this head-to-tail configuration (found in lines B73 and A636), *Helitron *No.1 would have excised cleanly (see next section), leaving only *Helitron *No.2 at the insertion site (as in McC, W22, and W23).

**Table 3:** Molecular Analysis of Two Adjacent *Helitrons*

**Inbred Line**	**F1 + R1**	**F3 + R3**	**F2 + R4**	**F4 + R4**	**Conclusions**
4Co63	x	x	N/A	1 kb	
A188	x	x	0.7 kb	N/A	No.1^-^No.2^-^
A636	3 kb	0.6 kb	x	x	No.1^+^No.2^+^
B73	3 kb	0.6 kb	x	1 kb	No.1^+^No.2^+^
BSSS53	x	x	N/A	N/A	
McC	0.5 kb	0.6 kb	x	0.7 kb	No.1^-^No.2^+^
CML139	x	x	0.7 kb	N/A	No.1^-^No.2^-^
H99	x	x	0.7 kb	x	No.1^-^No.2^-^
I137TN	x	N/A	N/A	1 kb	
Ki3	x	x	0.7 kb	N/A	No.1^-^No.2^-^
M14	x	x	0.7 kb	N/A	No.1^-^No.2^-^
Mo17	x	x	0.7 kb	N/A	No.1^-^No.2^-^
W22	0.5 kb	0.6 kb	x	N/A	No.1^-^No.2^+^
W23	0.5 kb	0.6 kb	N/A	N/A	No.1^-^No.2^+^

PCR results from different primer combinations.

^+^: *Helitron *present

^-^: *Helitron *absent

x: no PCR amplification

N/A: no PCR test

Conclusions were based on PCR results. Both No.1 and No.2 *Helitrons *were detected in lines A636 and B73, only *Helitron *No.2 in lines McC, W22, and W23, and neither *Helitron *in lines A188, CML139, H99, Ki3, M14, or Mo17. No conclusion could be reached for 4Co63, BSSS53, and I137 TN based on the above PCR results.

#### A Putative Helitron Somatic Excision

**Figure 9:**
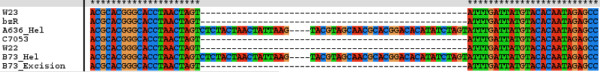
Alignment of Silico 3 sequences indicating the insertion of the predicted *Helitron *Silico3 in A636 and B73. There is no *Helitron *insertion in McC, W22, W23, or *bz-R*. It is interesting that, in addition to an occupied site, B73 also shows a weak Silico3 vacant-site-sized band in Fig. 4. Sequencing of this PCR product confirmed it to be an unoccupied site.

We further cloned and sequenced the PCR products of Silico3 from lines A636, B73, McC, W22, W23, and *bz-R*. Fig. 9 presents the sequence alignment showing the insertion of the predicted *Helitron *Silico3 in A636 and B73. There is no *Helitron *insertion in McC (C7053), W22, W23, or *bz-R*. The sequence results validate the HelitronFinder's prediction. It is interesting that, in addition to an occupied site, B73 also shows a weak Silico3 vacant-site-sized band (Fig. 6). Sequencing of this PCR product confirmed it to be an unoccupied site (Fig. 9). There are no sequence polymorphisms in the adjacent sequences to rule out the possibility that this band arose from DNA contamination in the B73 DNA preparation. Alternatively, however, this band may represent *Helitron *somatic excision products, which have been found at other polymorphic sites in maize (Y. Li and H.K. Dooner, unpublished data). This is a surprising result in light of the fact that *Helitrons *presumably transpose by a rolling circle transposition mechanism that does not generate empty sites.

#### Deletion of Helitron Flanking Regions

**Figure 10:**
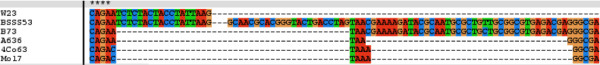
Alignment of Silico 1 sequences. Silico1 is present in W23 and BSSS63 and absent from B73, A636, 4Co63, and Mo17. The 3' flanking region in B73 is identical to that in BSSS53. However, the 3' end flanking regions of Silico1 in A636, 4Co63, and Mo17 are missing 38 nucleotides.

The PCR products of Silico1 (Fig. 5) from A636, B73, BSSS53, Mo17, W23, and 4Co63 were also cloned and sequenced. In addition to the BSSS53 inbred line from which Silico1 was predicted, we were able to amplify and sequence the 5' end of Silico1 from W23. The sequences of Silico 1 occupied and vacant sites are aligned in Fig. 10. Silico1 is present in W23 and BSSS63 and absent from B73, A636, 4Co63, and Mo17. The 3' flanking region in B73 is identical to that in BSSS53. However, the 3' end flanking regions of Silico1 in A636, 4Co63, and Mo17 are missing 38 nucleotides. The presence of the same deletion in three different lines points to a common origin of this chromosomal segment. Possibly, the deletion arose following the imprecise excision of Silico 1 from an occupied site in a common progenitor of these lines.

## Discussion

*Helitrons *are novel transposons that have not been well characterized experimentally. Implementing our maize *Helitron *discovery algorithm, we found two adjacent *Helitrons*, which we arbitrarily named No.1 and No.2, in the *r1 *region of B73 (Figs. 7 and 8). Here, we propose two models for how these adjacent *Helitron *arose. One hypothesis is that these are tandem repeats, which arose by the *Helitron'*s rolling circle mechanism of replication, as postulated by Pritham and Feschotte [9]. An alternative hypothesis is that one *Helitron *inserted next to an existing *Helitron*. The sequence data support the latter model. *Helitron *No.1 contains an S-receptor kinase gene with only one exon, whereas *Helitron *No. 2 carries an aldose reductase gene. We attempted to align these two *Helitrons*, excluding the S-receptor kinase and aldose reductase genes. There are large differences between the two *Helitrons*, indicating that *Helitrons *No. 1 and No. 2 do not represent tandem repeats. Our characterization of PCR products from several maize lines support the second hypothesis of two independent insertions, but the order of insertion is not clear. *Helitron *No. 2 could have inserted first, and No.1 subsequently, next to the 3' end of No.2, in which case No.1 would have inserted at a GT site, instead of the canonical AT site. Alternatively, the two *Helitrons *could have inserted in reverse order, followed by the precise excision of *Helitron *No.1 in a common progenitor of modern maize lines having only *Helitron *No. 2 at the insertion site.

Most known *Helitrons *in Table 1 carry gene fragments and not fully functional genes. One of the two adjacent *Helitrons *(No. 1) contains a gene with only one exon. We searched GenBank with both nucleotide and amino acid sequence queries and found a cognate single-exon gene in rice. This may indicate that *Helitron *No. 1 carries a fully functional gene. It is not clear at this point how *Helitrons *acquire host sequences, but it is important to learn if *Helitrons *have the ability to trap fully functional genes and mobilize them around the genome. More studies need to be conducted to determine if the gene inserted into *Helitron *No.1 is a fully functional gene.

We detected a putative *Helitron *excision product in the B73 inbred (Fig. 9), but could not rule out DNA contamination because of the absence of polymorphisms in the adjacent sequences. All four predicted *Helitrons *are present in some inbred lines and absent in others. This shows that *Helitrons *are active in the maize genome. We speculate that insertions and excisions of *Helitrons *can cause changes in the flanking regions, as the 38-bp deletion shown in Fig. 10.

## Conclusion

We have identified 140 new *Helitron *candidates in maize with our computational tool HelitronFinder. Four out of five candidates were confirmed to be real by empirical methods, thus validating the predictions of our program. Additional points to emerge from our study are that *Helitrons *may not always insert at an AT dinucleotide in the host sequences, that they can insert immediately adjacent to an existing *Helitron*, and that *Helitron *movement may cause changes in the flanking region, such as deletions.

## Methods

### Heuristic Search Algorithm of HelitronFinder

The HelitronFinder program is written in PERL and uses its regular expression abilities to look for the specified patterns of *Helitrons *in maize genome. The update_blastdb.pl script provided by NCBI was modified to work with the HelitronFinder program to download the maize genome DNA sequences in fasta file format when requested. The HelitronFinder will search the input DNA sequences from both forward and reverse directions. For each direction, there are two main subroutines to search for the 5' and 3' ends, respectively.

The 5' end subroutine uses the consensus derived from Figure 1 as its search criterion. This is relative straightforward. However, the 3' end structure is more complex, requiring a search for 16- to 20-bp palindromes in the DNA sequences. More specifically, we look for palindromes containing the self-pairing CG and the double A in the middle of the *HelA *type *Helitrons*. Then, the subroutine will identify 3' CTRR termini within 20 bp downstream of the palindrome and output the sequences from the beginning of the palindrome to the 3' CTRR terminus, along with their coordinates. For each possible instance of a 5' end, the subroutine lists the closest 3' ends within 50,000 bases.

The HelitronFinder program has two levels of constraints for the searching criteria, high quality and medium. The 5' end criterion of the high quality constraint is:

(TC [CT] [CA]TA [CT]TA [CA] [TC] [ATC] [ATC])([ATCG])([TA]TAAG)

The 3' end criterion of the high quality constraint is:

(CG)([ATCG]{3,5})(**AA**)([ATCG]{3,5})(CG)([ATCG]{9})(CTAGT)

The double 'A' in bold is one of the characteristics of *HelA *type *Helitron*. The high quality searching criterion is mainly targeting this type of *Helitrons*.

For the medium searching criterion, we use less constraints than the high quality criterion. The 5' end consensus is as close to the high quality as possible. However, we pick the less conserved 3' end as below:

(CG)([ATCG]{9,12})(CG)([ATCG]{1,13})(CT [AG] [AG]T)

This will be able to predict *HelB *type *Helitrons *as well.

### Primer Design

PCR primer pairs were designed based on the 500 bp of sequences flanking each *Helitron *end.

Silico 1 primers:

Forward CTGCACCACCGTCTCTACAA

Reverse TAGCCGCTCCTAAGAAGCAC

Silico 2 primers:

Forward GCGACCAAACCATAGCAAAA

Reverse AGGGGCATGAGTAGCTTCCT

Silico 3 primers

Forward1 (F1) CCACTTCTCCAGTTCCTTGG

Reverse1 (R1) GGGCGTAACATCATGTCATT

Forward2 (F2) GTTGGGACCCAGCTGTTAGA

Reverse2 (R2) ACCAAGAAGTTGGCCTCTCC

Forward3 (F3) AGGGTTTTCGTTGGAGGAGT

Reverse3 (R3) GATTCGAGTGTCCGCTTGAT

Forward4 (F4) AAGACAGCGGCTAGGGTTTT

Reverse4 (R4) TGTTTTGCACGGTGTGGTAG

Silico 4 primers

Forward TATCCCCGAGTCAAAACTGC

Reverse CGACGACAGCTTCACTGACA

### Cloning, Sequencing

PCR products then were cloned into pGEM-T easy vector (Promega). Sequences were obtained through 3700 DNA Analyzer using Big Dye v3.1 terminal reaction (Applied Biosystem). Consensus sequences were used for analysis.

## Availability and Requirements

The HelitronFinder program is available for public access at 

The detailed description and sample run are also provided at the website.

## Authors' contributions

CD conceived, designed and coordinated the study, carried out the sequence alignment and drafted the manuscript. JC implemented HelitronFinder in PERL. LH carried out the PCR and sequence analysis of the predicted *Helitrons *and helped to draft the manuscript. HKD designed and coordinated the study and helped to write the manuscript.
